# Ovarian Actinomycosis Mimicking Ovarian Malignancy: A Case Report

**DOI:** 10.7759/cureus.78194

**Published:** 2025-01-29

**Authors:** Nickolas Kintraia, Lali Barbakadze, Luka Katsitadze, Giorgi Javakhishvili, Platon Machavariani, Nato Metskhvarishvili, Maia Rizvadze, Eka Shvelashvili, Nino Khotivari, Natia Pkhaladze, Besik Japharidze, Tamar Didbaridze, George Burkadze

**Affiliations:** 1 Department of Obstetrics and Gynecology, Tbilisi State Medical University, Tbilisi, GEO; 2 Department of Obstetrics and Gynecology, Tbilisi State Medical University (TSMU) First University Clinic, Tbilisi, GEO; 3 Department of Microbiology, Tbilisi State Medical University, Tbilisi, GEO; 4 Department of Clinical Microbiology, Tbilisi State Medical University (TSMU) First University Clinic, Tbilisi, GEO; 5 Department of Pathology, Tbilisi State Medical University, Tbilisi, GEO

**Keywords:** actinomycosis, actinomycosis israelii, infectious disease medicine, ovarian actinomycosis, pelvic actinomycosis

## Abstract

*Actinomyces spp.* are non-spore-forming anaerobic bacteria that can be part of the normal flora of human oral, intestinal, and urogenital tracts. Mucosal disruption can lead to an infection characterized by granulomatous inflammation leading to abscess formation and sinus tracts classically draining pus with sulfur granules. Most actinomycosis cases are polymicrobial, involving various aerobic and non-aerobic bacteria. Cervicofacial actinomycosis is by far the most common, accounting for more than half of its presentations; however, cases of thoracic, abdominal, pelvic, and central nervous system (CNS) actinomycosis have also been described in the literature. Due to its non-specific symptoms, actinomycosis can mimic various conditions, including malignancies, Crohn’s disease, and tuberculosis. The diagnosis usually becomes clear after histopathologic examination. We would like to present a case of ovarian actinomycosis, a rare condition that has been associated with long-term intrauterine device (IUD) users and may mimic ovarian malignancy. Recognizing the patients at risk and using ancillary diagnostic testing may lead to early diagnoses and better outcomes for patients, including avoiding unnecessary surgery.

## Introduction

Actinomycosis is caused by a branching, filamentous, gram-positive bacteria from the *Actinomyces* genus. In humans, most infections are caused by *Actinomyces israelii *or *Actinomyces gerencseriae*; however, most cases tend to be polymicrobial [1]. Commonly identified co-infecting pathogens include *Aggregatibacter, Prevotella, Enterobacteriaceae, and Staphylococcus.*

*Actinomyces spp.* are part of the normal flora of the oral cavity, intestinal tract, and urogenital tract [2]. It is mostly an opportunistic pathogen that may cause disease after injury to a normal mucosal barrier. After the invasion, actinomycosis is characterized by a chronic, granulomatous response with tissue necrosis. The infection spreads contiguously and does not respect tissue planes. Over time, patients develop abscesses and sinus tracts draining pus and yellow sulfur granules that may be visible to the naked eye but are not always present.

Actinomycosis is a rare condition with an annual incidence of approximately 0.00003% [3]. Multiple risk factors have been identified, including mucosal trauma, immunosuppression, diabetes mellitus, and malnutrition. An intrauterine device seems to be the most consistent risk factor for pelvic actinomycosis. *Actinomyces* is part of a normal female genital flora, and a review of 20,000 Pap smears showed *Actinomyces*-like organisms in 0.26% of the smears. For intrauterine device (IUD) users, this number can go up to 7% [4]. Management of these women depends on their clinical presentations. For asymptomatic women with IUDs and A*ctinomyces*-like organisms identified on a Pap smear, the IUD is left in place. If there are symptoms concerning infection, the IUD should be removed and sent for culture, and the patient should be evaluated for pelvic inflammatory disease.

Culture and histopathologic testing are the keys to diagnosis. Matrix-assisted laser desorption ionization-time of flight mass spectrometry (MALDI-TOF MS) or 16S rRNA gene sequencing are newer ancillary methods that may allow for rapid and accurate identification of *Actinomyces spp.* but are not widely available [5,6].

## Case presentation

A 51-year-old female presented to the First University Clinic with malaise, loss of appetite, bloating, and aching pain in the right lower quadrant. She also noted one episode of defecation with bloody content. She was initially seen by a gastroenterologist, but the abdominal ultrasound and CT scan at the time were found to be normal. The complete blood count at the time was notable for anemia, as shown in Table 1.

**Table 1:** Laboratory results on admission revealing anemia and elevated inflammatory markers ESR: erythrocyte sedimentation rate

Parameters	Patient lab values	Normal lab values
Red blood cells	3.723 million/mm3	4.3-5.9 million/mm3
White blood cells	6.83 ×109/L	4.5 -11 × 109/L
Platelets	233 × 109/L	150-450 × 109/L
Hemoglobin	9.0 g/dL	12-16 g/dL
Hematocrit	26.7 L/L	36-44 L/L
ESR	50 mm/hr	<30 mm/hr

Due to unremitting symptoms, she was referred to the obstetrics and gynecology department. The patient’s history was significant for menopause onset two years ago and an 18-year history of IUD use, which was removed eight months ago. The patient noted that she first noted these symptoms after IUD removal.

A pelvic ultrasound revealed a normal-sized uterus with a subserosal node 33 x 31 mm in size, consistent with a uterine fibroid. The right ovary was found to have a cystic mass with thick hyperechogenic walls and non-homogeneous content. The cyst was found to be 86x75 mm in size, and the wall thickness was 2.2 mm. Cancer antigen-125 (CA-125) was noted to be normal at 14.92 U/mL.

**Figure 1:**
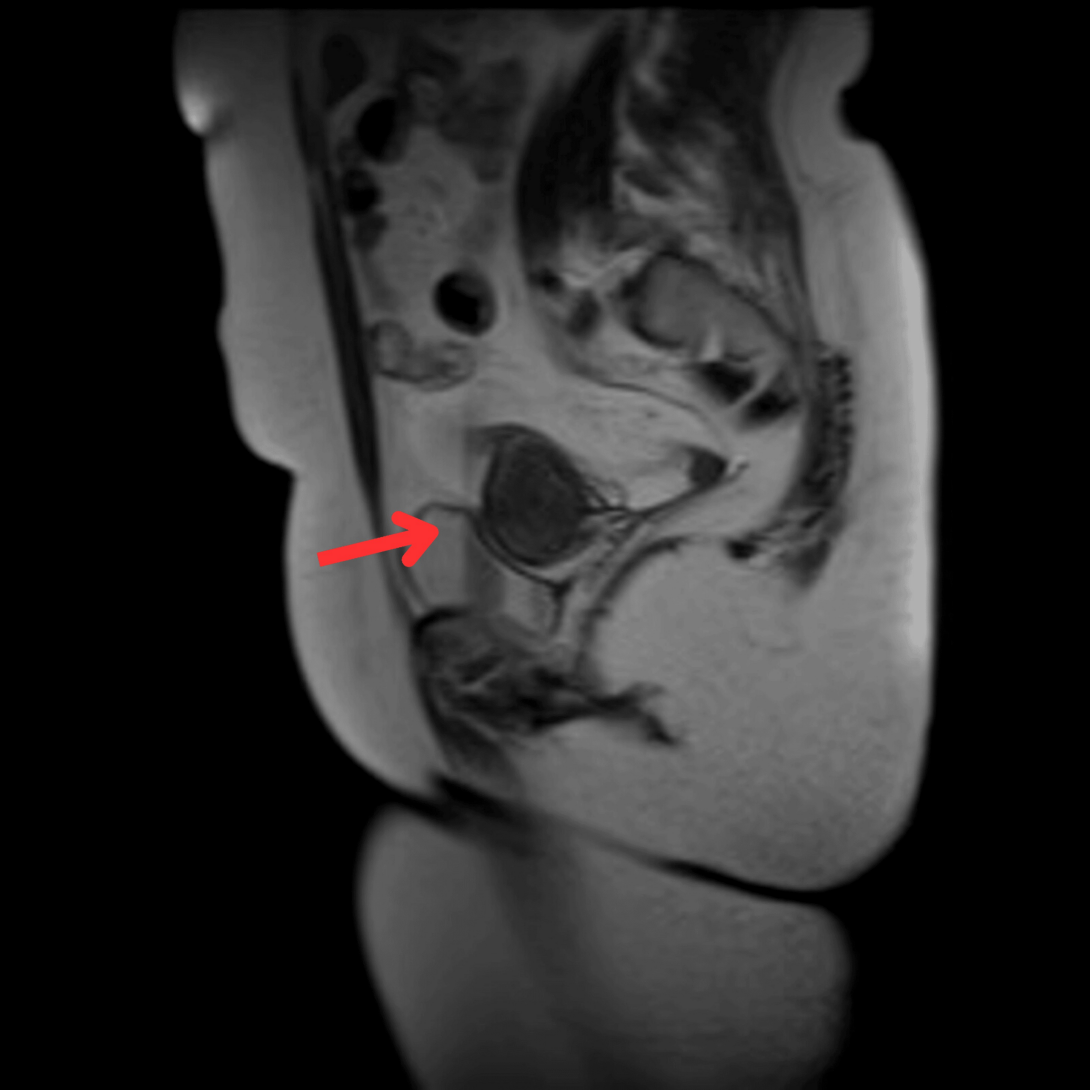
MRI showing right adnexal mass Arrow: adnexal mass

The patient was followed up with an MRI with contrast to further clarify the diagnosis. Imaging showed two cystic lesions and a 2.8x3.5 cm mass with contrast enhancement. The lymph nodes were found to be up to 1 cm in size bilaterally along the femoral vessels. The findings of the MRI are shown in Figure 1.

**Figure 2:**
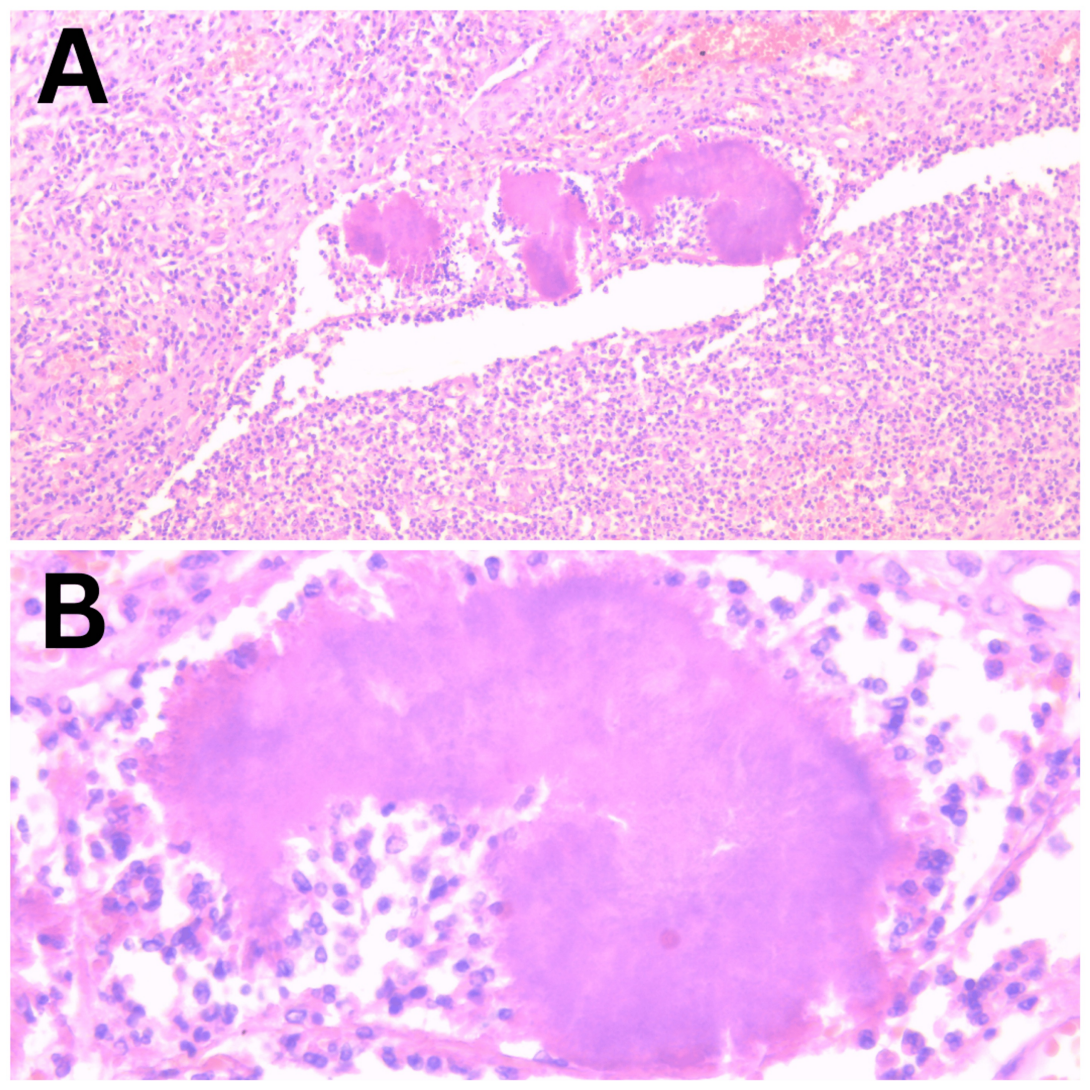
Histopathology of ovarian abscess A: prominent neutrophilic infiltrate, chronic granulomatous inflammation with aggregates of filamentous bacteria; B: classic "sulfur granules" of actinomycosis representing colonies of Actinomyces species.

The patient was diagnosed with an ovarian malignancy and underwent laparotomy with bilateral salpingo-oophorectomy and appendectomy. Intraoperatively, the uterus and the right adnexa were found in the conglomerate, and adhesions were noted along the omentum, ascending colon, and appendix. The contents of the right ovarian mass were found to be mucosal-hemorrhagic and cloudy. The contents were sent for culture, which were found to be negative. Histopathologic examination revealed neutrophilic infiltrate with branching filamentous bacteria, diagnosing ovarian actinomycosis. The findings of histopathologic examination are illustrated in Figure 2.

## Discussion

*Actinomyces spp. *can cause infections involving cervicofacial, pulmonary, abdominal, and pelvic regions. The infection is often non-specific and may mimic various other conditions, including malignancy. Because of this, diagnosis is often made postoperatively. Early diagnosis may help the patients avoid invasive procedures and lessen the emotional distress that comes with a cancer diagnosis. *Actinomyces spp.* have excellent sensitivity against penicillin antibiotics [7]. Although it is a rare infection, clinicians should be aware of the patients at risk. Mucosal trauma can allow bacterial invasion and the development of infection. In one study analyzing the results of cervical smears between 2011 and 2012 at a single university hospital, the incidence of *Actinomyces*-like organisms was found to be 0.26% (52/20,390). Around 42 of these women (80.8%) were intrauterine device users [4]. Non-specific symptoms of infection in an IUD user female should prompt actinomycosis as one of the differentials.

Cultures and histopathologic testing are the keys to diagnosis; however, the diagnosis is confirmed by culture in less than 50% of suspected cases [8].

The use of MALDI-TOF MS and 16S rRNA sequencing is emerging as promising techniques for *Actinomyces* identification. In a study done in 2018, the MALDI-TOF MS identified 68/77 (88.3%) of *Actinomyces* isolates to the genus level and 44/77 (57.1%) of *Actinomyces* isolates to the species level using the manufacturer's identification criteria [6].

## Conclusions

Ovarian actinomycosis is a rare but significant clinical entity that can mimic ovarian malignancy among other disorders, leading to unnecessary invasive procedures and significant patient distress. Clinicians should recognize the risk factors, such as prolonged IUD use, and maintain a high index of suspicion in patients presenting with non-specific infectious symptoms and an adnexal mass. Accurate diagnosis relies on clinical presentation, imaging, microbiological, and histopathologic testing. Emerging diagnostic methods such as MALDI-TOF MS and 16S rRNA sequencing offer promising avenues for the future. Increasing awareness of this infection can facilitate early intervention with appropriate antibiotic therapy, potentially avoiding unnecessary surgical procedures and improving patient outcomes.
